# Effect of Light on Cognitive Function During a Stroop Task Using Functional Near-Infrared Spectroscopy

**DOI:** 10.1007/s43657-021-00010-5

**Published:** 2021-04-19

**Authors:** Yafei Yuan, Guanghao Li, Haoran Ren, Wei Chen

**Affiliations:** 1 Department of Electronic Engineering, Center for Intelligent Medical Electronics, Fudan University, Shanghai, 200433 China; 2 Human Phenome Institute, Fudan University, Shanghai, 200433 China

**Keywords:** fNIRS, Stroop task, Light modulates, Cognitive function

## Abstract

Light modulates human brain function through its effect on circadian rhythms, which are related to several human behavioral and physiological processes. Functional near-infrared spectroscopy (fNIRS) is a noninvasive optical neuroimaging technique used for recording brain activation during task performance. This study aimed to investigate the effects of light on cognitive function, particularly in the prefrontal cortex using fNIRS. The effect of light on cognitive modulation was analyzed using the Stroop task, which was performed on 30 participants under three different light conditions (color temperature 4500 K, 2500 K, and none). The behavioral results indicated that light conditions can easily and effectively modulate the performance of tasks based on the feedback, including the response time and accuracy. fNIRS showed hemodynamic changes in the bilateral dorsolateral prefrontal cortices, and the activated brain regions varied under different light conditions. Moreover, light may be regarded as a safe, effective, inexpensive, and accessible tool for modulating human cognitive function.

## Introduction

Light modulates human brain function through virtue of its impact on circadian rhythms, which are related to several human behavioral and physiological processes (Vandewalle et al. 2011; Tam et al. 2016; Fisk et al. 2018). Consequently, light has been studied widely and is well demonstrated as an effective non-contact modulator of cognitive performance (Rao et al. 2016; Daneault et al. 2014; Hawes et al. 2012). Furthermore, light therapy is regarded as a safe, effective, and inexpensive modality for several clinical applications, such as improving sleep (Burkhalter et al. 2015), depression treatment (Terman and Terman 2014), mood disorder treatment (Maruani and Geoffroy 2019; Kuijsters et al. 2015), appetite adjustment (AlBreiki et al. 2015), glucose metabolism (Hirakawa et al. 2018) and improving cognition (Fisk et al. 2018; Forbes et al. 2014). The most profound function is improving cognition, which is involved in daily human activities. To be precise, the physical properties of light, such as intensity, color temperature, composition, timing, and the duration of exposure, influence its effects on cognitive performance (Rao et al. 2016). Hence, investigating the underlying interaction between light and human cognition is valuable to human health.

Recent studies have focused on investigating new cognitive neuroscience methods for exploring the constituent parts of the human cognitive system and its functional relationship with other mental processes (Fujiwara et al. 2015), especially functional neuroimaging methods (Mitchell 2008; Coltheart 2013). Functional near-infrared spectroscopy (fNIRS) is one of the noninvasive optical neuroimaging techniques used to examine brain activation during task performance (Manelis et al. 2019). Since 1977, when Jöbsis first proposed in vivo application of near-infrared spectroscopy (NIRS) (Jöbsis 1977), the technology has been adopted extensively for several applications (Quaresima and Ferrari 2019; Schaal et al. 2019). fNIRS involves the emission of near-infrared light into the scalp followed by the measurement of the intensity of reflected light. These measurements are used to calculate the relative concentrations of oxygenated hemoglobin (Oxy-Hb) and deoxygenated hemoglobin (Deoxy-Hb) separately using the modified Beer–Lambert law (Hoshi 2007; Hu et al. 2019a). fNIRS is relatively less expensive, more portable and tolerant of motion artifacts than other neuroimaging modalities. Additionally, because of its noninvasive nature, it can be used in the outpatient setting (Coltheart 2013; Hoshi 2007). It is widely employed to investigate human cognition (Curtin et al. 2019; Hu et al. 2019b; Holmes et al. 2019). The frontal areas of the brain, especially the prefrontal cortex (PFC), are associated with cognition (Xu et al. 2020; Rovetti et al. 2019; Moriarty et al. 2019). The PFC regions perform executive functions, such as higher-order cognitive functions, which are essential for planning and executing complex motor control actions (Udina et al. 2019; Ji et al. 2019; Schroeter et al. 2004). Hence, the PFC is considered as the region of interest (ROI) for investigating the relationship between light and cognition (Yang et al. 2019). Furthermore, studies on the application of fNIRS for investigating cognitive modulation by light are seldom reported.

This study aimed to investigate the effect of light on cognitive function using fNIRS particularly in the PFC. Focusing on how light modulates cognition, the participants were assessed under three different light conditions (color temperature 4500 K, 2500 K and none) using the Stroop task. The variation in Oxy-Hb in the ROIs with light was examined.

## Materials and Methods

### Participants

Thirty right-handed participants (13 males and 17 females) were selected for the study from the age group of 21–24 years. All participants had normal or corrected-to-normal vision and normal color vision. None of them had a history of neurological or psychiatric disorders or used psychotropic medications. The research protocol was approved by the Ethics Committee of Fudan University.

### Procedures and Materials

**Fig. 1:**
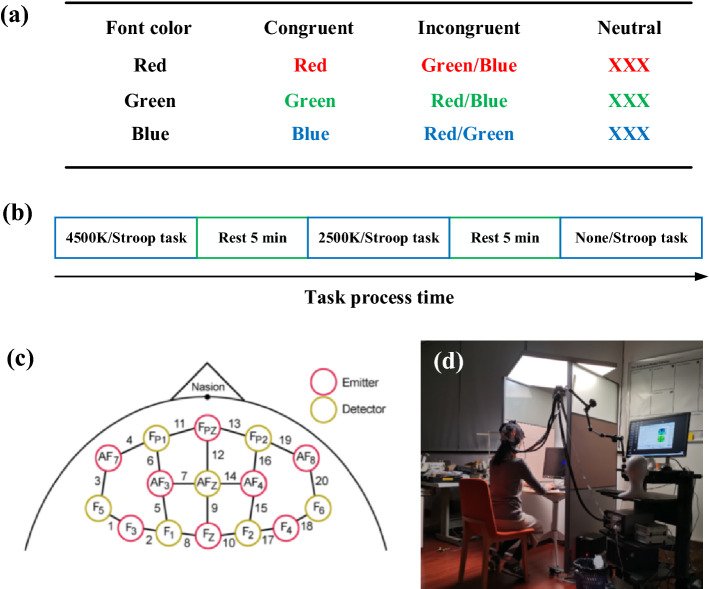
Task procedures and materials. **a** The color–word matching Stroop task rule; **b** the task process time; **c** the setting up of the fNRIS channels; **d** the actual testing arrangement

All participants performed three Stroop tasks under three different light conditions. The modified color–word matching Stroop task was used as an event-related cognition task in this research (MacLeod and MacDonald 2000; Yanagisawa et al. 2010a). The functional neuroimaging leveraged the Stroop task to explore human cognition (Ji et al. 2019; Yanagisawa et al. 2010a; Leon-Carrion et al. 2008; Byun et al. 2014). The rules for the single trial for the congruent, incongruent, and neutral conditions of the color–word matching Stroop task are shown in Fig. 1a. The single trial involved 12 words printed in black that appeared randomly. The complete task included 11 trials performed in one light mode. The participants were instructed to press the relevant response key during the executive task according to the rules. The participants needed to press the keys “1”, “2”, and “3” for the red, green, and blue color words, respectively. The response time (RT) and the response were recorded using a computer for further analysis.

The procedure for assessing the effect of light during a three-cycle Stroop task, is as shown in Fig. 1b.

The participants initially practiced the procedure with over 92% accuracy. The task process was as follows: Stroop task under the 4500 K color temperature → rest for 5 min → Stroop task under the 2500 K color temperature → rest for 5 min → Stroop task under no lighting → end.

The light source used in this research was fabricated using a light guide plate (LGP) with an embedded light emitting diode (LED), which prevents glare, visual fatigue, and improves visual effects (Pan and Fan 2011). The light source plane was fixed 1.2 m above the desk, as shown in Fig. 1d. Three light settings were designed for the research: 4500 K color temperature, 2500 K color temperature and no light. The desk illumination was approximately 320 lx.

### Data Acquisition by fNIRS

The fNIRS data were recorded using a multi-channel continuous-wave fNIRS system (NIRx Medical Technologies LLC-NIR-Scout, USA) consisting of eight LED light sources and eight photodetectors (Ji et al. 2019). The distance between the detector and the source was approximately 3 cm. The detector recorded relative changes in Oxy-Hb and Deoxy-Hb at a sample rate of 7.81 Hz at two wavelengths (760 and 850 nm) (Xu et al. 2019). The location of the probe and the arrangement of specific brain regions were similar to those of previous studies (Tsuzuki et al. 2007). In this study, we focused on the PFC for investigating cognition. Figure 1c shows the set-up of the fNRIS channels. Four different ROIs were selected: the right ventrolateral prefrontal cortex (R-VLPFC) (channels 17–20), the right dorsolateral prefrontal cortex (R-DLPFC) (channels 13–16), the left L-DLPFC (channels 5–7 and 11), and the posterior left L-VLPFC (channels 1–4). The fNIRS transmitters were wrapped tightly using a black bandage to ensure that there was no extraneous light interference during the Stroop cognitive task.

The participants were instructed to sit comfortably in a chair and maintain a calm and relaxed position. They were asked to focus on the screen with their minds blank. The visual task was presented on a 21-inch thin film transistor (TFT) screen.

### Data Processing

The fNIRS raw data were analyzed based on SPM with additional modules for ANOVA. First, a low-frequency band-pass filter (0.01–0.2 Hz) was applied to eliminate the baseline drift, artifact, and physiological noise. fNIRS records the changes in Oxy-Hb and Deoxy-Hb concentrations simultaneously. However, the selection of signals for analyzing the brain activation presents with some scientific challenges. In this research, we mainly focused on the Oxy-Hb signal changes, as the Oxy-Hb signal was observed to have a higher amplitude than the Deoxy-Hb signal (Xu et al. 2019). Furthermore, the signal-to-noise (S/N) ratio of Oxy-Hb is better than that of Deoxy-Hb and the signal is more sensitive for processing the task response (Cheng et al. 2015). The fNIRS channels were analyzed successively to record their activation during the Stroop task. If the light intensity of the channels fell below 400 mV or exceeded 4000 mV at any point during the session, they were excluded (Rovetti et al. 2019). The behavioral performance and the Oxy-Hb data were analyzed in Vision 22.0 SPSS using a 3 ((stimulus conditions) × (light condition)) repeated-measures ANOVAs.

## Results

### Behavioral Results: Stroop Interference

**Fig. 2:**
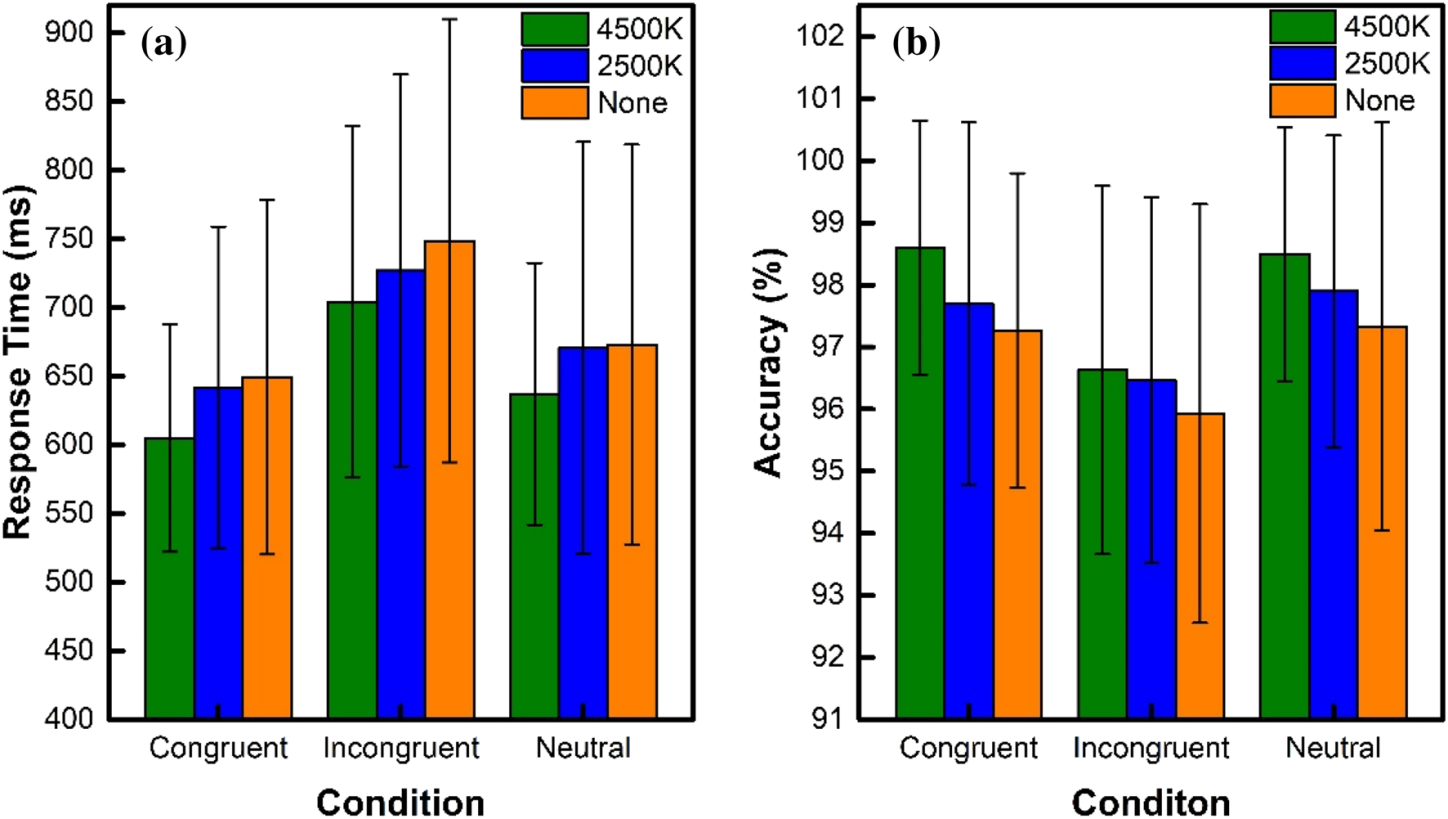
The Stroop task performance results. **a** Response times under three light conditions; **b** accuracies under three light conditions

The results of the Stroop tasks are shown in Table 1, including the RT and accuracy. Figure 2 shows the behavioral RT and the accuracies. Concerning the RT shown in Fig. 2a, by using the repeated-measure stimulus condition (congruent vs. incongruent vs. neutral) × light condition (4500 K vs. 2500 K vs. none), ANOVA demonstrated a significant effect for the stimulus condition (*F* = 46.81, *df* = 2, *P* < 0.0005) and light condition (*F* = 6.91, *df* = 2, *P* = 0.002). No significant effect of the interaction between the stimulus and light conditions was observed. All the stimulus conditions under different lights, such as 4500 K (*F* = 38.32, *df* = 2, *P* < 0.0005), 2500 K (*F* = 14.21, *df* = 2, *P* < 0.0005), and none (*F* = 24.44, *df* = 2, *P* < 0.0005) showed an obvious Stroop interference. Further, all the light conditions with different stimulus conditions, such as congruent (*F* = 4.79, *df* = 2, *P* = 0.012), incongruent (*F* = 4.13, *df* = 2, *P* = 0.021), and neutral (*F* = 3.26, *df* = 2, *P* = 0.046) were also significantly different. The RT of the incongruent stimulus condition was significantly longer than those of the congruent and neutral stimulus conditions under all three light conditions. The participants responded more quickly under the 4500 K light condition than under the other two light conditions.

**Table 1:** Stroop task performance results

Lighting set-up	Response time (ms)			Accuracy (%)		
	Congruent	Incongruent	Neutral	Congruent	Incongruent	Neutral
4500 K	605.0 ± 82.9	704.3 ± 127.8	637.0 ± 95.4	98.6 ± 2.0	96.6 ± 3.0	98.5 ± 2.0
2500 K	641.9 ± 117.0	727.1 ± 142.8	670.6 ± 150.0	98.0 ± 2.7	96.5 ± 3.0	97.9 ± 2.5
None	649.4 ± 129.0	748.7 ± 161.5	673.0 ± 145.6	97.6 ± 2.7	95.9 ± 3.4	97.5 ± 3.4

Regarding the accuracy shown in Fig. 2b, using the repeated-measure stimulus conditions (congruent vs. incongruent vs. neutral) $\times$ light conditions (4500 K vs. 2500 K vs. none), ANOVA demonstrated a significant effect of the stimulus condition (*F* = 7.32, *df* = 2, *P* = 0.001) and light condition (*F* = 4.25, *df* = 2, *P* = 0.019). The effect of the interactions between the stimulus and light conditions was not observed. This suggests that the accuracy was higher under the 4500 K light condition than that under the two other light conditions. Furthermore, the accuracies were significantly lower under the incongruent condition than the congruent and neutral conditions. The lowest accuracy was recorded under the no light condition in all three stimulus conditions. The participants showed the best behavioral performance under the 4500 K light condition. This performance was not only better based on the RT, but also the accuracy. This study showed that the 4500 K light condition provides an efficient and friendly environment.

## fNIRS Results: Brain Activation

**Fig. 3:**
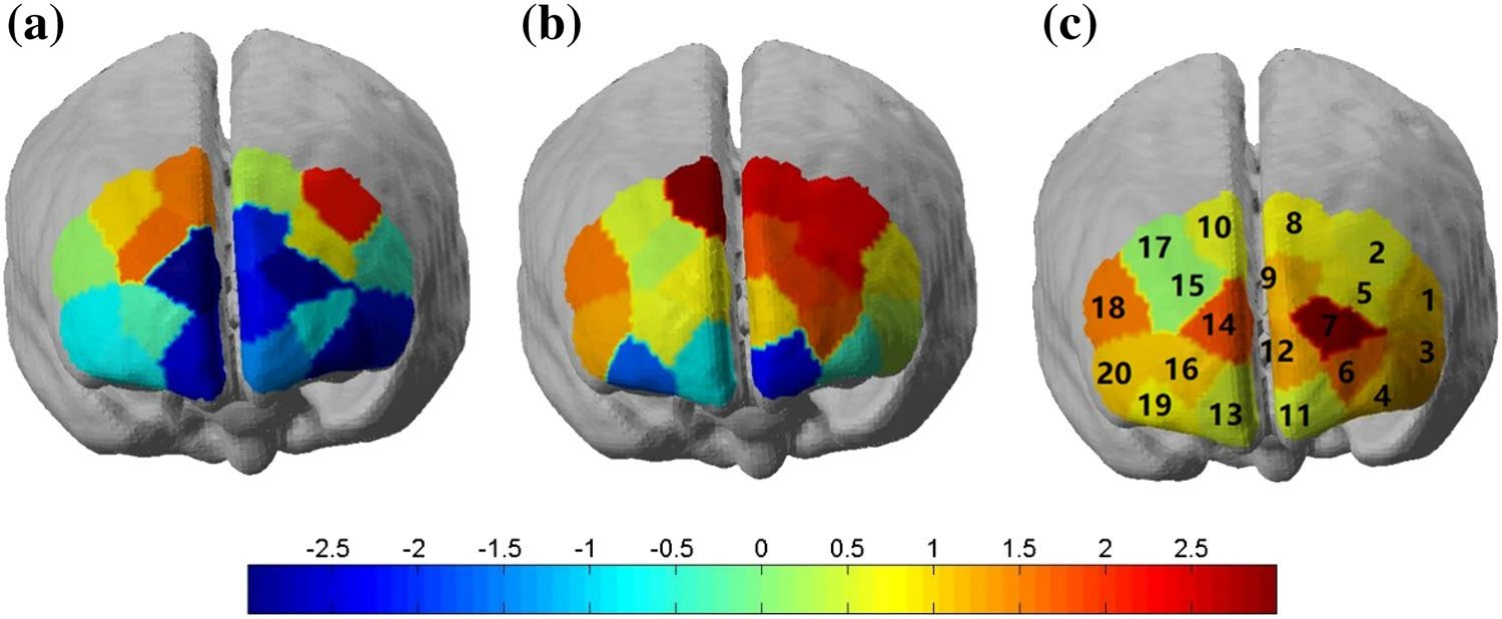
The brain activations under the different light conditions during the Stroop task. **a** The brain activation under the 4500 K light condition; **b** the brain activation under the 2500 K light condition; **c** the brain activation under the no-light condition

Figure 3 shows the t-statistic map for the brain activation by Oxy-Hb under three light conditions: 4500 K, 2500 K, and none. Figure 3a shows the brain activation by the Oxy-Hb in the channels (2,10, and 15) mainly located in the VLPFC under the 4500 K light condition. Figure 3b shows the brain activation corresponding to the Oxy-Hb concentrations in the channels (2, 5, 8, 10, and 18) mainly located in the VLPFC under the 2500 K light condition. Figure 3c shows the brain activation corresponding to the Oxy-Hb concentration in the channels (1, 3, 6, 7, 9, 12, 14 and 18) mainly located in the VLPFC and DLPFC under the no-light condition. A significant difference was observed in the brain activations of the PFC under the three light conditions. The lowest mean Oxy-Hb concentration was recorded under the 4500 K light condition during the Stroop task. However, the no-light condition required the highest mean Oxy-Hb concentration to complete the task. Moreover, when comparing the three brain activation modes, the relevant ROI changes in response to the variations in the light conditions were observed. The main changes in the mean Oxy-Hb concentration under the 2500 K light condition were observed in the R-VLPFC and L-VLPFC. The changes in the mean Oxy-Hb concentration under the no-light condition were mainly observed in the ROIs, as compared with the 4500 K light condition.

**Fig. 4:**
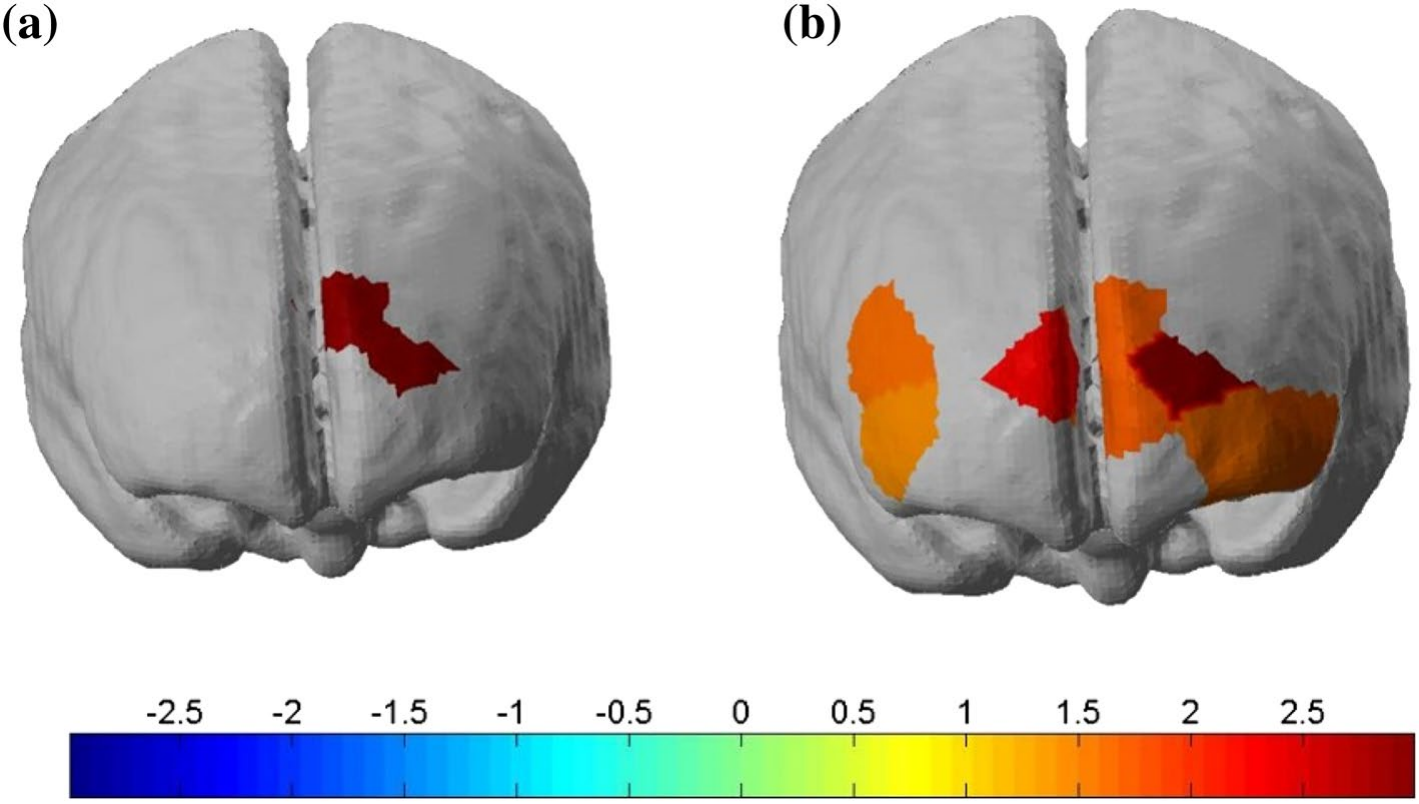
The significant difference between the Oxy-Hb concentrations of the channels. **a** The comparison of the Oxy-Hb concentrations under the 2500 K and 4500 K light conditions; **b** the comparison of the Oxy-Hb concentrations under the no-light condition and the 4500 K light condition.

**Fig. 5:**
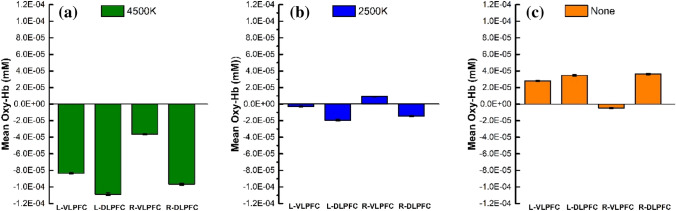
Mean Oxy-Hb concentration in ROIs under three light conditions. **a** Mean Oxy-Hb concentration in ROIs under the 4500 K light condition; **b** mean Oxy-Hb concentration in ROIs under the 2500 K light condition; **c** mean Oxy-Hb concentration in ROIs under the no-light condition

To locate the exact channel variates, we used a paired t-test to statistically compare the PFC activations under different light conditions. The results for all the channels with their significant differences (*P* < 0.05) are shown in Fig. 4. Figure 4a shows that the channels (6 and 7) have significant differences under the 4500 K and 2500 K light conditions. Figure 4b shows that the channels (3, 4, 6, 7, 9, 12, 14, 18, and 20) present significant differences under the 4500 K and the no-light conditions. To investigate the variates and hemodynamic responses associated with the worsening light conditions, we evaluated the mean Oxy-Hb concentration of the ROIs, including the L-VLPFC, L-DLPFC, R-VLPFC, and R-DLPFC. Figure 5 illustrates the mean Oxy-Hb concentrations in the ROIs under the three light conditions. Significant differences were observed between the Oxy-Hb concentrations of the ROIs under the three light conditions. The mean Oxy-Hb concentrations in the L-VLPFC, L-DLPFC, and R-DLPFC increase as the light conditions worsen.

## Discussion

**Fig. 6:**
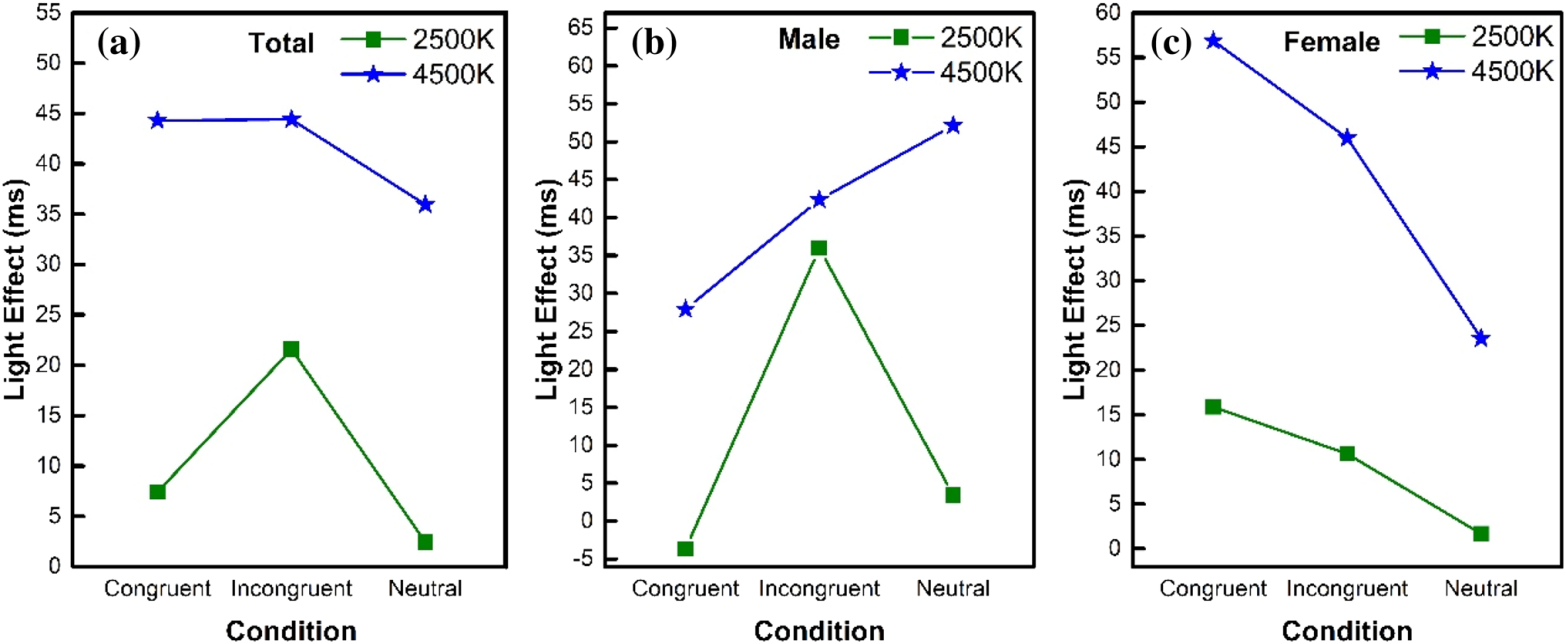
The effects of light on behavioral performance. **a** Overall effects of light on behavioral performance; **b** effects of light on behavioral performance in males; **c** effects of light on behavioral performance in females

The present study aimed to investigate the effect of light conditions on cognitive function and PFC brain activation during the color–word matching Stroop task. Stroop interferences existed under all three light conditions as reported in the literature (MacLeod and MacDonald 2000). The assessments of behavioral performance under the light and dark conditions showed improved function under the light condition. Moreover, this improvement in function was higher under the 4500 K than the 2500 K light condition. Figure 3 illustrates that channels for the activation of the functional areas of the brain were increased to complete the cognitive task as the light conditions worsened. In other words, the 4500 K light condition required fewer channels to complete the task, as the brain was relaxed. Figure 4 illustrates the significant difference (*P* < 0.05) between the channels for the 4500 K and 2500 K light conditions, as well as the 4500 K light condition and the no-light condition. The Oxy-Hb concentration increased during the processing from the 4500 K light condition to the no-light condition. The highest Oxy-Hb concentration was observed under the no-light condition, whereas the lowest Oxy-Hb concentration was observed under the 4500 K light condition. The results were based on the RT and accuracy. The participants performed faster with a higher accuracy under the 4500 K light condition. This indicates that people can be more proficient in friendly light environments. The effects of light on behavioral performance stratified by the light conditions are shown in Fig. 6. As shown in Fig. 6a, the effect of light under the 4500 K condition was higher than that under the 2500 K condition for all the stimulus conditions, which had more facilitative effects on the executive functions, resulting in a faster response and high accuracy. Interestingly, a difference in the effects of light was observed between the male and female participants, particularly under the incongruent stimulus condition. The male participants performed similarly when exposed to the incongruent stimuli under the two light conditions, whereas the female participants had significantly different responses.

Additionally, the fNIRS results showed the Oxy-Hb concentrations in response to the Stroop task in the bilateral VLPFC and DLPFC. The apparent hemodynamic changes were detected in the bilateral DLPFC regions under different light conditions, which are consistent with previous studies on cognition by event-related neuroimaging (Ji et al. 2019; Leon-Carrion et al. 2008; Byun et al. 2014). Under the efficient and friendly work light environment (4500 K), the VLPFC was activated mainly by increasing the Oxy-Hb concentrations of three channels (2,10, and 15). As the light conditions worsened, the VLPFC was activated. More specifically, the 2500 K light condition was associated with channels 2, 5, 7, 8, 9, 10 and 18, and the dark condition was associated with channels 1, 3, 6, 7, 12, 14 and 18. Previous studies have concluded that the activation of the right DLPFC may be due to its sensitivity to the difficulty of the task (Newman et al. 2003). A similar phenomenon was observed in our study, as the Oxy-Hb concentration of channel 14 increased under the dark conditions. The highest Oxy-Hb concentration was recorded in channel 7 located at the L-DLPFC. This region is related to the neural substrate, which is responsible for improving Stroop performance (Yanagisawa et al. 2010b). As the light environment darkened or worsened, channel 7 in this region was activated. Based on the behavioral and fNIRS results, it was concluded that the light conditions could effectively modulate human cognitive function. It is proposed that humans require lower Oxy-Hb concentrations and show higher work improvements under the 4500 K light condition. It is noteworthy that humans have worse performance and require high Oxy-Hb concentrations under the no-light condition. The lower Oxy-Hb consumption means it is easy for humans to fulfill tasks. Compared with the lower color and darker light conditions, this research demonstrates that appropriate light condition make people more productive. In summary, this study confirmed that light can be regarded as a safe, effective, inexpensive, and accessible tool for modulating human cognitive function. Additionally, fNIRS is a reliable method for exploring the mechanisms underlying the brain activation for cognitive performance in relation to relevant factors.

## Conclusion

This study investigated the PFC changes associated with cognitive functions. This study focused on the effect of light on human cognition and explored the underlying mechanisms. The behavioral results indicated that light conditions can easily and effectively modulate task performance, and this was based on feedback information, such as RT and accuracy. It was also proposed that working under an appropriate light condition can improve work efficiency; worse light conditions impair performance. fNIRS detected hemodynamic changes in the bilateral DLPFC regions and the activated brain regions varied under the different light conditions. Furthermore, it was observed that light is a sensitive modulator of cortical hemodynamics, which is associated with cognitive function. The two results indicate that humans require lower Oxy-Hb concentrations and show better performance and improved work efficiency under the 4500 K light condition. It is also noteworthy that humans show worse performance and require high Oxy-Hb concentrations under worse light conditions. Moreover, light can be regarded as a safe, effective, and accessible tool for modulating human cognitive function, which is of significance for the application of light therapy in the future.
